# Corrigendum to “Comprehensive assessment of estrogen receptor beta antibodies in cancer cell line models and tissue reveals critical limitations in reagent specificity” [Mol. Cell Endocrinol. 440 (2016) 138–150]

**DOI:** 10.1016/j.mce.2017.01.048

**Published:** 2017-03-05

**Authors:** A.W. Nelson, A.J. Groen, J.L. Miller, A.Y. Warren, K.A. Holmes, G.A. Tarulli, W.D. Tilley, B.S. Katzenellenbogen, J.R. Hawse, V.J. Gnanapragasam, J.S. Carroll

**Affiliations:** a Cancer Research UK Cambridge Institute, University of Cambridge, Robinson Way, Cambridge, CB2 ORE, UK; b Academic Urology Group, Department of Surgery, University of Cambridge, Cambridge, CB2 0QQ, UK; c Department of Urology, Addenbrooke's Hospital, Cambridge University Hospitals NHS Foundation Trust, Hills Road, Cambridge, CB2 0QQ, UK; d Department of Histopathology, Cambridge University Hospitals NHS Foundation Trust, Hills Road, Cambridge, CB2 0QQ, UK; e Dame Roma Mitchell Cancer Research Laboratories, Hanson Institute Building, School of Medicine, Faculty of Health Sciences, The University of Adelaide, SA 5005, Australia; f Departments of Molecular and Integrative Physiology, University of Illinois at Urbana-Champaign, Urbana, IL 61801, USA; g Department of Biochemistry and Molecular Biology, Mayo Clinic, 200 1st Street SW, Rochester, MN 55905, USA

The authors regret that some of the information in Table 2 is incorrect. The correct text should read:

CWK-F12 Recombinant fusion protein. Amino acids 256–505 of human wt ERb Mouse Monoclonal C-terminus WB, IP, IHC.

The authors would like to apologise for any inconvenience caused.

